# Early clinical experiences with nintedanib in three UK tertiary interstitial lung disease centres

**DOI:** 10.1186/s40169-017-0172-3

**Published:** 2017-11-03

**Authors:** Hannah Toellner, G. Hughes, W. Beswick, M. G. Crooks, C. Donaldson, I. Forrest, S. P. Hart, C. Leonard, M. Major, A. J. Simpson, N. Chaudhuri

**Affiliations:** 1 Manchester Medical School, University of Manchester, Stopford Building, Oxford Rd, Manchester, M13 9PT UK; 2 Manchester University NHS Foundation Trust, Wythenshawe Hospital, Southmoor Rd, Wythenshawe, Manchester, M23 9LT UK; 3 Hull and East Yorkshire Hospitals NHS Trust, Castle Road, Cottingham, HU16 5JQ UK; 4 Newcastle Upon Tyne Hospitals NHS Trust, Queen Victoria Rd, Newcastle upon Tyne, NE1 4LP UK

**Keywords:** Idiopathic pulmonary fibrosis, Nintedanib, Treatment, Real world

## Abstract

**Background:**

Nintedanib has been shown to slow disease progression in patients with idiopathic pulmonary fibrosis (IPF). It was approved by the National Institute for Health and Care Excellence (NICE) in January 2016 for IPF patients with a forced vital capacity (FVC) of 50–80% in the United Kingdom (UK).

**Aim:**

To report real world data about our early clinical experience using nintedanib in 187 patients with a multi-disciplinary (MDT) diagnosis of IPF in a manufacturer funded patient in need scheme (three UK centres) prior to NICE approval.

**Methods:**

All patients with a MDT diagnosis of IPF from December 2014 to January 2016 commenced on nintedanib were included. Demographic details, adverse events (AEs) and where available lung function results were retrospectively collected from clinical letters.

**Results:**

187 patients (76% males) with a median age of 72 years (49–89) were treated with nintedanib. The average pre-treatment FVC was 81.1 ± 19.8% and diffusion capacity of the lungs for carbon monoxide was 43.9 ± 15% (n = 82). Fifty percent of patients started nintedanib because they were ineligible for pirfenidone due to an FVC > 80%. The median treatment course was 8 ± 4 months. The majority of patients experienced 1–3 AEs with nintedanib (52%, n = 97). The most frequent AEs were diarrhoea (50%), nausea (36%), reduced appetite (24%), tiredness (20%) and gastro-oesophageal reflux (18%). The majority of AEs resulted in no change in treatment (64%, n = 461). 21% (n = 150) of AEs resulted in a dose reduction and 13% (n = 94) necessitated discontinuation of treatment. 1 in 5 patients discontinued treatment either temporarily or on a permanent basis during the monitoring period. In a select cohort of patients, a statistically significant greater proportion of patients remained stable or improved and a lower proportion declined, as depicted by FVC changes of > 5% after nintedanib commencement (P < 0.05 using Chi squared test).

**Conclusions:**

Nintedanib is well tolerated and has an acceptable safety profile. Only 8% of those reporting diarrhoea discontinued treatment either on a temporary or permanent basis. There were no signals with respect to increased cardiovascular morbidity or major bleeding risk. This is in keeping with the INPULSIS clinical trial findings but in a real world cohort.

## Background

Idiopathic pulmonary fibrosis (IPF) is a chronic progressive interstitial lung disease (ILD) of unknown aetiology with a 3–4 year median survival rate after diagnosis [1]. The prognosis is worse than most malignancies other than lung and pancreatic cancer [2]. Adults of a median age of 70 years are most commonly affected and present with progressive dyspnoea and cough with or without sputum. There is great heterogeneity in disease course. Commonly there is a step wise reduction in lung function over time with accumulating morbidity which leads to end stage respiratory failure [3]. Some patients may suffer from acute exacerbations of IPF. These are episodes of decline in lung function, without identifiable cause, which might represent periods of acceleration of lung damage and have a high rate of morbidity and mortality [4]. Until recently, lung transplantation was the only treatment proven to improve survival in IPF patients. This intervention is not available to a large proportion of this patient group [5]. Two novel agents: nintedanib and pirfenidone have now been shown to slow the decline in lung function in clinical trials [6–9].

The first clinical trial with nintedanib identified that a dose of 150 mg twice a day over a period of 12 months slowed the decline in FVC in IPF patients [An early phase two trial: ‘To Improve IPF with BIBF’ (TOMORROW)]. There was a 68.4% reduction in the rate of annual FVC decline compared to placebo in this patient group. Moreover, this dose of nintedanib also decreased the frequency of acute exacerbations (2.4 vs 15.7 per 100 patient-years, P = 0.02) [6].

Two further phase two randomised controlled trials (RCT), INPULSIS I and II carried out over 52 weeks enrolling a total of 1066 patients, provided compelling evidence that nintedanib is effective at slowing FVC decline compared to placebo. The INPULSIS-1 trial showed that the annual rate of change in FVC was − 114.7 ml with nintedanib compared to − 239.9 ml with placebo (P < 0.001) and − 113.6 to − 207.3 ml in INPULSIS-2 (P < 0.001). However, the effect on time to first acute exacerbation was inconsistent across both studies. Neither trial was powered to assess mortality effect [7].

The most commonly reported adverse events (AEs) identified in the INPULSIS studies were gastrointestinal and classified as mild to moderate in nature. Diarrhoea occurred with the highest frequency in patients allocated to the treatment group (INPULSIS-1: 61.5% vs 18.6% placebo, INPULSIS-2: 63.2% vs 18.3%). The overall proportion of patients with serious AEs was comparable in both treatment and placebo groups. Nintedanib was discontinued in 25.2% of patients in the treatment group vs 17.6% in the placebo group in INPULSIS-1. In INPULSIS-2 23.7% patients discontinued their treatment compared to 20.1% of the placebo group. These discontinuations were most often related to an AE. It was noted that temporarily reducing the dose or discontinuing the drug temporarily had no significant effect on the overall rate of FVC decline [7].

There is limited data regarding clinical experiences with nintedanib in the real world, in patients who are older, with co-morbidities and who may not fit the stringent criteria required for clinical trial acceptance. The German Compassionate Use Program (CUP) and patient in need (PIN) scheme in the UK facilitated earlier treatment with nintedanib, prior to licensing and widespread manufacturer availability and offered the means to collect real world data for IPF patients. Bonella et al. followed up 62 patients who were enrolled into the CUP in nine German centres over a mean period of 8 ± 4 months. The majority had been previously treated with pirfenidone. Most patients FVC stabilised on treatment within 6 months, defined as < 5% change from baseline (63%). In common with the clinical trials, gastrointestinal symptoms were most commonly identified in 67% of patients. Diarrhoea was experienced by 63% of patients, anorexia by 39%, nausea by 26% and weight loss in 50% [10]. The proportion of patients with weight loss was higher than reported in the clinical trials and is also reported in a Spanish study of 20 patients, where this figure was 40% [11]. No new safety concerns were identified. We therefore aimed to provide further experience on the real world utilisation of nintedanib for IPF.

## Methods

### Data collection

This is a multi-centre retrospective observational study, formed by members of the Northern Interstitial Lung Disease Network: Manchester University NHS Foundation Trust (MFT), Hull and East Yorkshire Hospitals NHS Trust and Newcastle Upon Tyne Hospitals NHS Trust.

All IPF patients seen at the three UK centres who were started on nintedanib as part of the patient in need scheme, prior to NICE approval, between December 2014 and January 2016 were included (n = 187). The patient inclusion criteria included patients with a MDT diagnosis of IPF, those who were ineligible for pirfenidone treatment due to an FVC less than 50% or greater than 80% or who suffered from intolerable AEs on pirfenidone.

Data were collected retrospectively from clinical letters and pulmonary function test (PFT) results from beginning of treatment to June 2016 at MFT and beginning of treatment to January 2016 at the other centres. The monitoring period at MFT was 18 months compared to 10 months in Hull and 2 months in Newcastle. We focused on demographic information, reasons for starting nintedanib, frequency and type of AEs whilst on treatment, discontinuation rates and pre-treatment lung function results for all centres. Serious AEs were defined as ‘any adverse event that resulted in death, was immediately life-threatening, resulted in persistent or clinically significant disability or incapacity, required hospitalization, or was deemed serious for any other reason’ [7].

Where available we collected additional details including serial lung function data, smoking history, use of ambulatory or long term oxygen therapy (LTOT) and shuttle walk distance for selected patients from the coordinating centre for this study (MFT).

Clinical letters were written by ILD doctors or specialist nurses after each outpatient clinic appointment. They were stored electronically on the patient health record and sent to the general practitioner. Patients were followed up on a monthly basis by an ILD doctor or specialist nurse for 6 months and then three monthly thereafter. A letter was sent for each clinic appointment. At the initial appointment patients were counselled about common AEs. Further follow up appointments involved clarification of current treatment dose, identification and management of AEs and other significant health problems, repeat baseline blood tests and PFTs at intervals of 6 months. This information was noted and transcribed onto each letter.

### Treatment regimen

Patients had baseline bloods taken prior to commencing treatment. These included a full blood count (FBC), liver function tests (LFTs), urea and electrolytes (U and Es) and PFTs. Eligible patients were prescribed nintedanib 150 mg tablets twice daily to be taken 12 h apart with food.

### Statistical analysis

The data were analysed using Microsoft Excel. Continuous normally distributed variables are presented as median (range) and non-continuous variables as mean ± standard deviation. Significance of lung function results was calculated using the Chi squared test. P < 0.05 was significant and stabilisation of lung function was defined as FVC change < 5% predicted 12 months after treatment initiation.

## Results

### Demographics

187 patients were included in the study (124 MFT; 51 Hull; 12 Newcastle), of which 76% were male. The median age was 72 ± 8 years with a range spanning 49–89 years. The average pre-treatment predicted FVC was 81.1 ± 19.8% (n = 187) and pre-treatment DLCO was 43.9 ± 15% (n = 82).

The median length of treatment was 282 days (range 12–492) at MFT, 272 days (range 6–457) at Hull and 28 days (range 28–56) at Newcastle. We collected additional information regarding shuttle walk distance, smoking status and use of LTOT from the MFT patient cohort only. This is represented in Table 1.

**Table 1:** Shuttle walk distance, smoking status and oxygen use of MFT patients (n = 124)

Shuttle walk n = 40	Distance
Most recent shuttle walk distance	397 m
Average pre-walk oxygen saturation	96%
Average post-walk oxygen saturation	87%
Smoking status n = 124	Number (%)
Ex-smoker	83 (67)
Non-smoker	35 (28)
Current smoker	3 (2)
Unknown	3 (2)
Average pack years	33
Ambulatory and/or long term oxygen therapy n = 124	43 (35)

#### Nintedanib is most frequently started because FVC is greater than 80%

The reasons for starting nintedanib in all centres were as follows:

- An FVC greater than 80% predicted in 94 patients (50%).
- An FVC less than 50% predicted in 12 patients (6%).
- Intolerable AEs on pirfenidone in 38 patients (20%).
- Refusing pirfenidone based on AE profile in 32 patients (17%).
- Decline in FVC more than 10% on pirfenidone in 4 patients (2%).
- Skin carcinoma in 3 patients (2%).
- Estimated glomerular filtration rate (eGFR) < 30% in 3 patients (2%).
- Unknown in 1 patient (1%).

#### Adverse events are common and mainly gastrointestinal with nintedanib

**Fig. 1:**
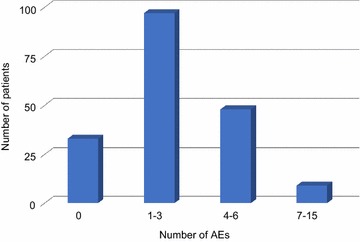
The total number of patients experiencing adverse events

A total of 723 AEs were reported in 187 patients from all centres, which corresponds to an average 3.9 AEs per individual patient. During the study period 18% of patients reported no AEs (n = 33), where five patients had no record of any follow up data. 52% reported 1–3 AEs (n = 97) and 26% of patients reported 4–6 AEs (n = 48). 5% reported 7–15 AEs (n = 9) (Fig. 1).

AEs per patient were retrospectively collected as described in “Methods”. The data is depicted as the total number of patients experiencing AEs ranging from a frequency of 0–15 (n = 187).

The most frequent AEs as a percentage of the total events are represented in Table 2. The percentage of patients who experienced each AE is also shown alongside results from the INPULSIS trials for comparison [7]. The most frequent AEs were diarrhoea (26%), followed by nausea (14%), reduced appetite (9%), tiredness (8%) and gastro-oesophageal reflux (GOR) (7%). Abnormal liver function tests accounted for 3% of the total number of AEs (n = 20). 49.7% of patients experienced at least one episode of diarrhoea (n = 93). Conversely, the proportion of patients who complained of nausea and reduced appetite in our study was 36.4% (n = 68) and 23.5% (n = 44) respectively. Serious AEs made up 5% of the total number of AEs; death (2%, n = 18), nose bleeds, haematemesis, haemoptysis (2%, n = 16), myocardial infarction (MI) (< 1%, n = 2) and pulmonary embolism (PE) (< 1%, n = 1).

**Table 2:** Frequency of adverse events in comparison to the INPULSIS I and II clinical trials

Adverse event (AE)	Reported events	Percentage of total events (n = 723), %	Percentage of patients experiencing AE (n = 187), %	Percentage of patients experiencing AE from INPULSIS-I, % [7]	Percentage of patients experiencing AE from INPULSIS-II, % [7]
Diarrhoea	185	26	49.7	61.5	63.2
Nausea	102	14	36.4	22.7	26.1
Reduced appetite	65	9	23.5	8.4	12.8
Tiredness	60	8	20.3	–	–
GOR	53	7	18.2	–	–
Abdominal pain, bloating and wind	58	8	24.1	–	–
Weight loss	29	4	14.4	8.1	11.2
Vomiting	21	3	8.6	12.9	10.3
Abnormal elevated LFTs	20	3	9.6	4.9	5.2
Death	18	2	9.6	–	–
Constipation	12	2	4.3	–	–
Nose bleeds, haematemesis, haemoptysis	16	2	7	–	–
Cough	10	1	2.7	15.2	11.6
Taste disturbance	9	1	3.7	–	–
Headache	7	1	3.2	–	–
Mood disturbance	7	1	2.7	–	–
Aching joints	5	1	2.1	–	–
Chest discomfort	5	1	0.5	–	–
Bronchitis	5	< 1	2.7	11.7	9.4
AKI	4	1	1.6	–	–
Abnormal blood count results	3	< 1	1.6	–	–
Rash	3	< 1	1.1	–	–
Increased dyspnoea	3	< 1	1.1	7.1	8.2
Sleep disturbance	3	< 1	1.1	–	–
Dizziness	2	< 1	1.1	–	–
Recurrent urinary tract infections (UTIs)	2	< 1	0.5	–	–
MI	2	< 1	0.5	1.6	1.5
Pruritis	3	< 1	1.6	–	–
Other viral/bacterial infection	2	< 1	1.1	–	–
Mouth pain/swelling	2	< 1	1.1	–	–
Unknown	2	< 1	1.6	–	–
PE	1	< 1	0.5	–	–
Hair loss	1	< 1	0.5	–	–
Anosmia	1	< 1	0.5	–	–
Thigh/calf ache	1	< 1	0.5	–	–
Hypersalivation	1	< 1	0.5	–	–

The most frequent AEs as a percentage of the total events are represented (n = 723). The percentage of patients who experienced each AE (n = 187) is also shown alongside results from the INPULSIS trials for comparison [7].

#### The majority of adverse events with nintedanib can be managed by either no change in treatment or dose reduction

**Fig. 2:**
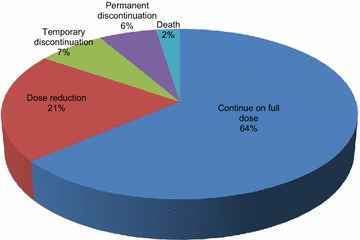
The impact of each individual adverse event on nintedanib treatment

A total of 723 AEs were recorded in 187 patients. Overall, 64% of all AEs resulted in no change in treatment (n = 461) and 21% led to a reduction in dose (n = 150). Whereas, only 7% of AEs resulted in temporary discontinuation (n = 51) and 6% led to permanent discontinuation (n = 43) (Fig. 2).

Data is depicted as the action taken with nintedanib for each individual adverse event that was experienced as a percentage of the total number of AEs (n = 723).

Diarrhoea was the most common AE reported with nintedanib. No change in treatment was reported for 71% of these AEs (n = 132). Only 8% of those reporting diarrhoea discontinued treatment either on a temporary or permanent basis (n = 15). Overall it was the most common AE leading to a change in treatment course.

**Fig. 3:**
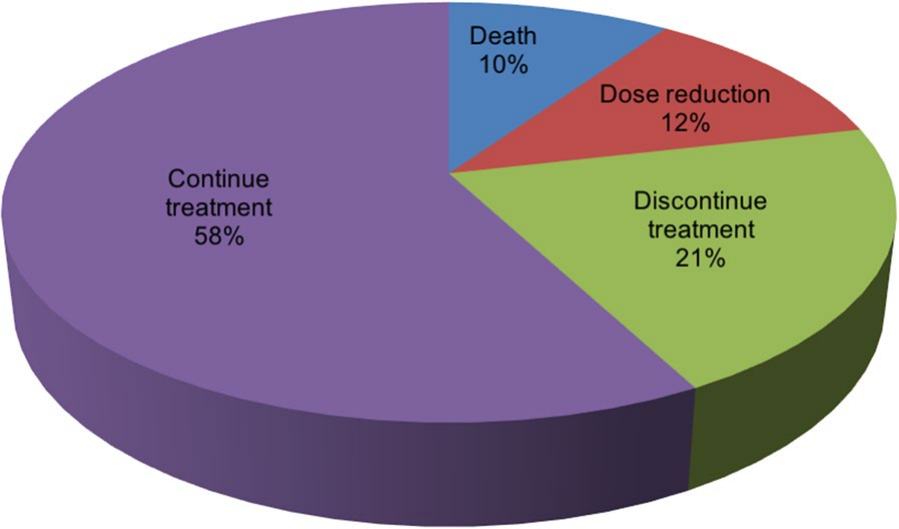
The impact of adverse events on nintedanib treatment at the end of the monitoring period

At the end of the monitoring period, treatment was discontinued in 21% of patients (n = 39). 16% stopped permanently (n = 30) and 5% paused treatment temporarily (n = 9). 58% of patients continued treatment at the full dose (n = 108), 12% were taking a reduced dose (n = 22) and 10% had died (n = 18) (Fig. 3).

Data is depicted as a percentage of the total patient population (n = 187).

#### Lung function remained stable or improved for a select cohort of patients

**Fig. 4:**
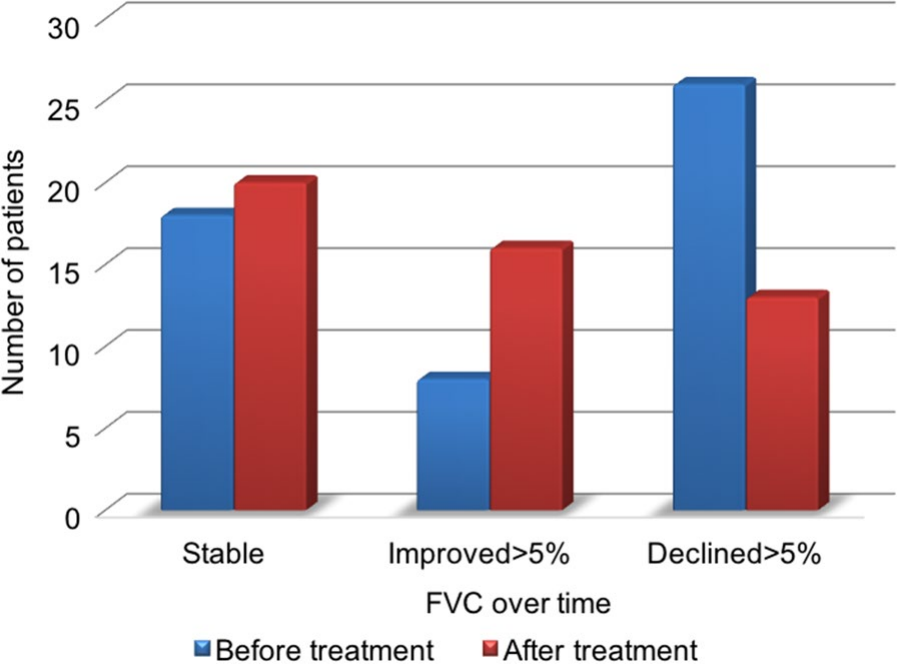
The impact of nintedanib treatment on lung function

Serial lung function data were collected for 124 patients at MFT. Lung function results before treatment (both − 12 and − 6 months) were available for 42% (n = 52) of patients and 39% (n = 48) of patients after treatment (both + 6 and + 12 months). A statistically significant greater proportion of patients remained stable or had improvement in lung function after treatment with nintedanib compared to before treatment. Likewise a lower proportion of patients had an FVC decline of greater than 5% after treatment with nintedanib (P < 0.05 using Chi squared test) (Fig. 4).

FVC lung function data were available in a cohort of patients 12 months before (n = 52) and 12 months after (n = 48) nintedanib treatment. Serial measurements over 12 months allowed patients to be depicted as stable [minimal (0–4%) or no change in FVC over time], improved (improvement in FVC > 5% over time) or declined (decline in FVC > 5% over time). Data is depicted as the total number of patients that remained stable, improved or declined as defined above, before and after nintedanib treatment.

## Discussion

Nintedanib was initially developed as an anti-angiogenic drug to treat cancer. It targets the same enzymes identified in the cell signalling cascades of IPF. These are pro-angiogenic intracellular tyrosine kinase enzymes; receptors for fibroblast growth factor, vascular endothelial growth factor and platelet derived growth factor [12]. Early tumour models have shown that nintedanib is ‘highly active at well tolerated doses’ and has sustained pathway inhibition kinetics affecting three cell types: pericytes, smooth muscle cells and endothelial cells [12]. Nintedanib also has other broader downstream targets including non-receptor tyrosine kinases of the Src family, which have pro-fibrotic activity [13, 14]. Beyer et al. postulated that there was an increased risk of AEs due to the action of nintedanib blockading multiple pathways [13]. In vitro models of IPF have indicated that nintedanib has anti-fibrotic activity by inhibition of the cellular processes of fibroblasts and myofibroblasts and possible contributing angiogenesis [14]. The efficacy of nintedanib at improving lung function has also been shown in a recent IPF mouse model, indicating that it not only reduces fibrosis but also significantly enhances the pulmonary microvasculature [15]. This prior research gives a strong basis for the clinical efficacy of nintedanib in humans. It also indicates the need for careful monitoring of potential AEs.

The results of this retrospective real world study involving three UK tertiary IPF centres show that nintedanib has an acceptable AE profile and most AEs (85%) can be managed without treatment discontinuation. 723 AEs were identified in 187 patients, corresponding to 3.9 AEs per patient. AEs were common in patients prescribed nintedanib and the majority reported 1–3 AEs over the monitoring period. The most frequent AEs were diarrhoea (26%), followed by nausea (14%), reduced appetite (9%), tiredness (8%) and GOR (7%).

The type of AEs reported in our study also reflects many of those commonly identified in the INPULSIS trials. Diarrhoea was the most frequently identified AE in all studies, including our own, the clinical trials, the CUP trial and Spanish study [7, 10, 11]. Approximately half of our patients reported an episode of diarrhoea whilst on treatment, compared to 61.5 and 63.2% patients in INPULSIS I and II [7]. It was also the AE most likely to lead to a change in treatment course i.e. dose reduction or treatment discontinuation. Patients registered in the clinical trials reported significantly more episodes of cough and nasopharyngitis than in our study. We present a higher frequency of reduced appetite, nausea and abnormal LFTs compared to the clinical trials. The proportion of patients who complained of nausea and reduced appetite in our study was 36.4% (n = 68) and 23.5% (n = 44) respectively. These figures were 10% higher than reported by both INPULSIS studies. Our study also identified a higher number of patients with abnormal LFTs than in the clinical trials (9.6% vs 4.9% and 5.2%). This trend is mirrored in the results from the CUP trial where 8% of patients had an ALT/AST increase of more three times the upper limit [10]. We did not identify an increased proportion of patients with weight loss compared to the INPULSIS clinical trials.

The differences in AE reporting may be in part due to under or over reporting of symptoms as a consequence of the bias that is involved in the unblinded nature of real-world data collection. This includes researcher and reporter bias, with variation in reporting between clinicians and documentation of AEs in clinic letters. Our patient cohort is also older and have more comorbidities, as outlined in our British Thoracic Society (BTS) IPF registry data (unpublished data), than those enrolled in clinical trials. We have 451 of the 1119 (41%) patient records on the BTS-IPF registry. At presentation 81% of patients have comorbidities, the commonest being hypertension (30%), ischaemic heart disease (23%), diabetes (20%) and gastroesophageal reflux (10%). As a result of these factors and the presence of concomitant medication the AE profile is likely to differ. It is also possible that there is a degree of cross-over whereby progressive symptoms of IPF are attributed as an AE. Reassuringly, serious AEs represented only a very small number of the total number of AEs in our study (5%). These included pulmonary embolism and myocardial infarction in three patients of our total cohort. The INPULSIS trials did not show data for all individual serious AEs in their final analysis, aside from MI [7]. Our proportion of patients with MI was lower than identified in the INPULSIS trials. We therefore conclude that there is no increased signal of MI in the real world compared to the clinical trial environment. This is consistent with the results from the INPULSIS-ON open label single arm extension study [16].

There were 16 AEs associated with various degrees of bleeding (haemoptysis, haematemesis and nose bleeds), which corresponds to 2% of the total number of AEs. Only two of the patients in our study were prescribed concomitant anticoagulation or dual anti-platelet therapy. This was primarily due to the constraints of the manufacturer product information for nintedanib listing anticoagulation and high dose platelet use as a contraindication to starting nintedanib. The majority of bleeding events resulted in no change in treatment (50%, n = 8) with some leading to temporary discontinuation (38%, n = 6). Since nintedanib appears to have a significant effect on remodeling of the lung microvasculature, further clear guidance is necessary for patients taking both dual anti-platelet therapy or anticoagulation and nintedanib.

Demographic details for patients in our study and the INPULSIS trials were similar [7]. 76% of our patients were male compared to 81 and 78% in INPULSIS 1 and 2. Where data was available, 67% of a selected cohort of patients had previously smoked which is similar to the 70 and 66% of patients classed as ex-smokers in the clinical trials. In general, our patients were older with a mean age of 72 ± 8 years compared to 66.9 ± 8.4 and 66.4 ± 7.9 in the INPULSIS clinical trials. Our patients also had a wider range of co-morbidities which may have excluded them from clinical trial enrolment. It is therefore encouraging that despite these differences our patient group had similar experiences with no new AE signals in the real world setting.

The pre-treatment FVC of patients was 79.5 ± 17 and 80 ± 18.1 in both clinical trials [7]. This compares with an average FVC of 81.1 ± 19.8% in our study. The higher FVC in our study was primarily driven by the inclusion criteria for the patient in need program. This program was reserved for patients who were not eligible for pirfenidone due to the stringent UK NICE criteria of requiring an FVC between 50 and 80% to commence pirfenidone. The majority of patients in the PIN program therefore had an FVC of greater than 80%. However, despite this the average DLCO was 44% representing significant disease burden.

### Management of AEs

It is clear that patients often experience AEs with nintedanib however it is encouraging that most of these are tolerated by patients. 64% of all reported AEs in our study were successfully managed with no change to treatment regimen and 21% by dose reduction. In fact, 71% cases of diarrhoea led to no change in treatment at all and only 8% resulted in discontinuation. Similarly, the INPULSIS trials showed a less than 5% discontinuation rate with nintedanib because of diarrhea [7]. This figure was 11% in the CUP trial [10]. This reflects that AEs can usually be managed adequately without impacting long term treatment adherence.

The measures taken to alleviate AEs in both our real world study and the INPULSIS trials were similar. Patients could reduce their dose to 100 mg twice daily or temporarily stop treatment while the AE resolved. At the end of the monitoring period in our study, 22 patients (12%) were taking a reduced dose, which included those on 100 mg twice a day and also some who were taking 100 mg in the morning and 150 mg in the evening. These are patients where a balance needs to be achieved between optimum treatment of their IPF and quality of life. A reduced dose may lessen the severity of some AEs but it is as yet unclear whether this has any effect on slowing disease progression. The INPULSIS trials only identified that a dose of 150 mg twice daily reduces decline in lung function [7]. Post hoc analysis is emerging that a dose of 100 mg of nintedanib may be effective in reducing FVC decline however more research is needed to understand the best options for these patients [17].

In our study patients were also counselled to take the nintedanib with food to help with symptoms of nausea and were commonly prescribed loperamide for diarrhoea. Proton pump inhibitors, H2 histamine receptor antagonists and anti-emetics were also concomitantly advised for some patients.

### Discontinuation rates

By the end of the study period, nintedanib treatment was permanently discontinued in 21% of all patients, compared to 25.2% in INPULSIS-I and 23.7% in INPULSIS-II [7]. The slightly lower rate of discontinuation in our study is reassuring given the older age, wider range of lung function values and co-morbidities of our patient group. The lower rate of discontinuation in our study might be explained by the increased overall treatment and follow up time compared to the clinical trials. It is however higher than the 11% of patients who were identified to have permanently stopped the medication as part of the CUP study [10].

This study was limited by biases that are introduced due to unblinded real world data documentation that is not as robust as that of clinical trials. The monitoring period times of the different centres varied from 2 months in Newcastle for 12 patients to 18 months for 124 patients at MFT according to when the PIN program was commenced. This limits the quality of the AE data. Some of our data is incomplete and biased towards those patients who attended regular follow up appointments and who survived for longer periods of time. The significance of the lung function results may be biased towards those with less severe disease and those that survived. We did not impute lung function values for patients who had died. These problems are representative of the limitations of collecting retrospective data in the real world compared to the robustness of clinical trials.

## Conclusions

In conclusion, our real world data focusing on AEs and their impact on treatment with nintedanib, is similar to the findings of INPULSIS clinical trials. This is one of the largest to date real world cohort studies of nintedanib use in IPF patients. We have shown AEs are common but that nintedanib has an acceptable AE profile. No new serious safety concerns have been identified, nintedanib is tolerated by the majority of patients and a higher proportion of a select cohort of patients had an FVC which remained stable or improved on treatment.

## Abbreviations

IPF: idiopathic pulmonary fibrosis
ILD: interstitial lung disease
NICE: The National Institute for Health and Care Excellence
UK: United Kingdom
AE: adverse event
MFT: Manchester University NHS Foundation Trust
MDT: multi-disciplinary team
FVC: forced vital capacity
PIN: patient in need scheme
CUP: compassionate use program
TOMORROW: To Improve IPF with BIBF
LTOT: long term oxygen therapy
DLCO: diffusing capacity of the lungs for carbon monoxide
eGFR: estimated glomerular filtration rate
FBC: full blood count
LFTs: liver function tests
U and Es: urea and electrolytes
PFTs: pulmonary function tests
AKI: acute kidney injury
GOR: gastro-oesophageal reflux
MI: myocardial infarction
UTI: urinary tract infection
PE: pulmonary embolism
